# Identification of a Candidate IgE‐Binding Maize Protein in a Patient With Corn‐Dependent Exercise‐Induced Anaphylaxis: A Case Report

**DOI:** 10.1155/crii/2731365

**Published:** 2026-10-19

**Authors:** Ayako Goto, Nobue Takamatsu, Tatsuya Moriyama, Yasuto Kondo, Shinichiro Nagamitsu

**Affiliations:** ^1^ Department of Pediatrics School of Medicine Fukuoka University Fukuoka Japan fukuoka-u.ac.jp; ^2^ Department of Food and Nutrition Beppu University Oita Japan; ^3^ Department of Applied Cell Biology Graduate School of Agriculture Kindai University Nara Japan kindai.ac.jp; ^4^ Department of Pediatrics Fujita Health University Bantane Hospital Nagoya Japan

**Keywords:** buckwheat allergy, corn allergy, food-dependent exercise-induced anaphylaxis, globulin-1S, maize protein, sequence similarity

## Abstract

Corn‐dependent exercise‐induced anaphylaxis (food‐dependent exercise‐induced anaphylaxis [FDEIA]) is rare, and its causative allergens remain poorly defined. Here, we report a case of corn‐dependent FDEIA and investigate candidate IgE‐binding proteins using immunological and proteomic analyses. A 13‐year‐old boy with a history of buckwheat allergy developed recurrent anaphylaxis during exercise after ingesting corn‐containing foods. An exercise challenge after ingesting 272 g of sweet corn reproduced the symptoms and confirmed the diagnosis. Corn‐specific IgE was 3.72 UA/mL, and skin prick testing was positive. IgE immunoblotting revealed patient‐specific bands at ~45 and 60 kDa. Two‐dimensional immunoblotting and mass spectrometry identified globulin‐1S (GLB1) and other proteins as candidate IgE‐binding proteins in this patient. Sequence analysis revealed significant local sequence similarity between maize GLB1 and buckwheat Fag e 5 within the second cupin domain. These findings identify GLB1 as a candidate IgE‐binding protein in this patient with corn‐dependent FDEIA. Although sequence similarities were observed between maize GLB1 and buckwheat Fag e 5, their immunological and clinical significance remains unclear, and further studies are required to determine whether cross‐reactivity exists.

## 1. Introduction

Food‐dependent exercise‐induced anaphylaxis (FDEIA) is a distinct form of food allergy, in which symptoms occur only if food ingestion is combined with exercise [1]. Although wheat is the most commonly reported trigger, corn‐related FDEIA is rare, and its allergenic components remain poorly characterized [2].

Currently, only a limited number of cases of corn‐dependent FDEIA have been reported with heterogeneous findings regarding specific IgE levels, skin test results, and identified allergenic proteins. Previous studies have suggested several candidate proteins, including granule‐bound starch synthase I (GBSS I); however, consistent allergenic components remain undetermined [3].

These findings highlight the incomplete understanding of maize allergens involved in FDEIA.

This study describes a case of corn‐dependent FDEIA and investigates candidate IgE‐binding proteins using immunological and proteomic approaches, with additional sequence analysis of maize and buckwheat proteins.

## 2. Case Presentation

A 13‐year‐old boy with a known history of buckwheat allergy presented with recurrent episodes of anaphylaxis triggered by exercise after ingesting corn‐containing foods. His buckwheat‐specific IgE level was >100 UA/mL, and he was diagnosed with a buckwheat allergy during infancy.

Since 6 years of age, he has experienced repeated episodes of cough, dyspnea, and facial angioedema during exercise after consuming sweet corn, popcorn, or foods cooked with sesame oil. Based on the patient’s clinical history, corn‐dependent FDEIA and sesame allergy were suspected.

Oral food challenge with sesame induced an immediate reaction, indicating that a simple food allergy was due to sesame. An exercise challenge test was performed after ingesting 272 g of sweet corn according to the Bruce protocol. Approximately 5 min after initiating the exercise, the patient developed facial edema and dyspnea. Symptoms resolved within 1 h of the oral administration of the antiallergic medication. These findings confirmed a diagnosis of corn‐dependent FDEIA.

Corn‐specific IgE was 3.72 UA/mL, and skin prick testing was positive. The patient tolerated corn ingestion without symptoms in the absence of exercise.

For proteomic analysis, corn protein extracts were prepared from commercially available popcorn because the patient had previously developed dyspnea during exercise after ingestion of popcorn. ELISA using popcorn extracts demonstrated significantly higher reactivity in the patient serum compared with controls, with dose‐dependent responses, suggesting specific IgE reactivity to popcorn‐derived proteins. Proteins were then extracted, purified, and separated by SDS‐PAGE under reducing conditions, followed by IgE immunoblotting using the patient serum (Figure 1). Immunoblot inhibition assays were also performed using a crude popcorn extract (0.1 mg/mL) as the inhibitor. Two‐dimensional immunoblotting and mass spectrometry were performed by Hoyu Co., Ltd. (Aichi, Japan).

**Figure 1:**
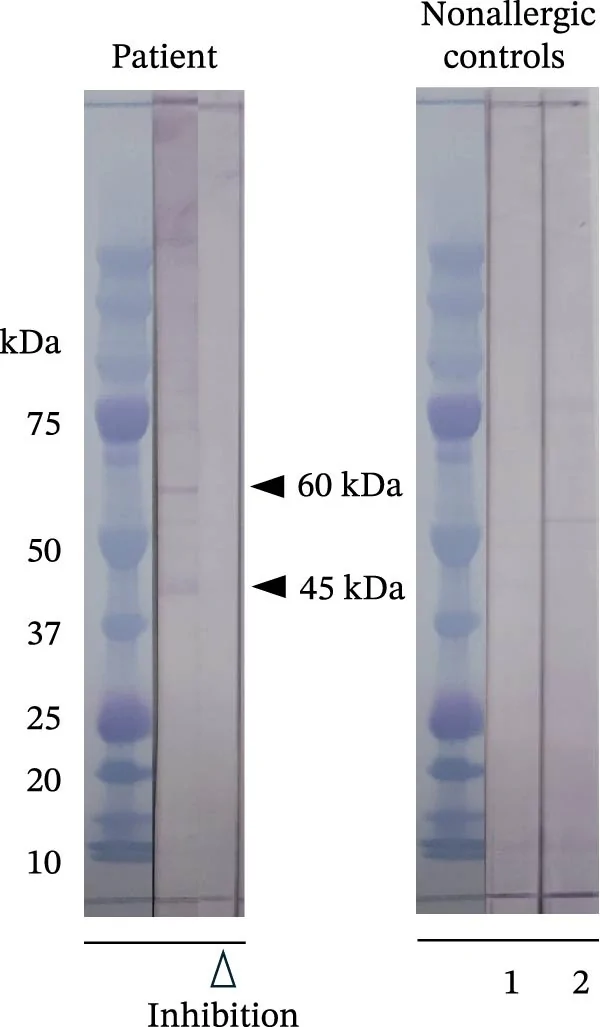
IgE immunoblotting of corn proteins. SDS‐PAGE and IgE immunoblotting were performed using the corn protein extracts under reducing conditions. The patient’s serum showed specific IgE‐reactive bands at ~45 and 60 kDa (filled arrows). No reactivity was observed in the sera of the two nonallergic control individuals (lanes 1 and 2). Immunoblot inhibition was performed using crude popcorn extract (0.1 mg/mL), resulting in significantly reduced IgE binding in the inhibition lanes (open arrows), confirming the specificity of the detected bands. The molecular weight markers (kDa) are shown.

IgE immunoblotting revealed patient‐specific bands at ~45 and 60 kDa. Two‐dimensional immunoblotting was performed to further characterize the IgE‐reactive proteins (Figure 2). Mass spectrometric analysis identified three proteins from the patient‐specific IgE‐reactive spots: globulin‐1S allele (GLB1; PWZ58485.1), ketol‐acid reductoisomerase, chloroplastic (PWZ10042.1), and UDP‐glucose pyrophosphorylase 2 (ONM57090.1). Their identification scores (ProteinPilot Unused scores) were 13.19, 26.07, and 51.41, respectively, and their sequence coverages were 15.6%, 13.6%, and 70.6%, respectively.

**Figure 2:**
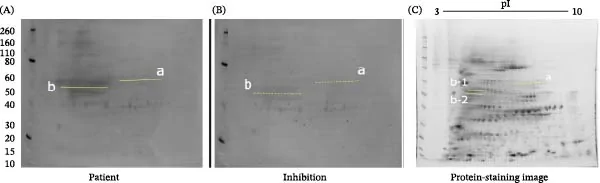
Two‐dimensional immunoblotting and identification of candidate IgE‐binding proteins. (A) Immunoblotting without inhibition. Solid lines (a and b) indicate patient‐specific IgE‐reactive signals identified by two‐dimensional immunoblotting. (B) Immunoblot inhibition using crude popcorn extract (0.1 mg/mL). Dashed lines indicate that the signals (a and b) were reduced or abolished, confirming the specificity of the patient‐derived IgE reactivity. (C) Protein‐staining image corresponding to the two‐dimensional immunoblot. Spot a was identified as globulin‐1 S allele (GLB1) by mass spectrometry. The IgE‐reactive region designated as spot b, located at ~50–60 kDa, was separated into spots b‐1 and b‐2 based on the corresponding protein‐staining pattern. Mass spectrometry identified ketol‐acid reductoisomerase, chloroplastic in spot b‐1 and UDP‐glucose pyrophosphorylase 2 in spot b‐2. The ~45‐kDa IgE‐reactive band observed in the one‐dimensional immunoblot could not be unequivocally matched to a specific spot in the two‐dimensional immunoblot.

## 3. Discussion

Here, we describe a case of corn‐dependent FDEIA in which several candidate IgE‐binding proteins were identified by immunological and proteomic analyses. Although FDEIA is a well‐recognized form of food allergy, the allergens responsible for non‐wheat triggers remain poorly characterized. In particular, only a limited number of cases of corn‐dependent FDEIA have been reported, and the responsible IgE‐binding proteins have not been consistently identified.

IgE immunoblotting revealed patient‐specific IgE‐reactive bands at ~45 and 60 kDa. Two‐dimensional immunoblotting followed by mass spectrometric analysis identified three proteins from patient‐specific IgE‐reactive spots: globulin‐1S allele (GLB1), ketol‐acid reductoisomerase, chloroplastic, and UDP‐glucose pyrophosphorylase 2. Notably, UDP‐glucose pyrophosphorylase 2 was also identified, together with GBSS I, as a potential IgE‐binding protein in a previously reported patient with corn‐dependent FDEIA [3]. The identification of UDP‐glucose pyrophosphorylase 2 in both the present and previously reported cases is noteworthy and suggests that its potential role in corn‐dependent FDEIA warrants further investigation. However, the present findings alone do not establish any of these proteins as causative allergens.

Among the three proteins identified in the present patient, we focused on GLB1 for further exploratory analysis based on its structural and biochemical characteristics. This selection was hypothesis‐driven and was not based on the relative intensity of the IgE‐reactive signal or the protein identification score. GLB1 belongs to the 7S globulin family within the cupin superfamily, whose members typically contain two cupin domains and form trimeric structures in their native state [4–6]. A globulin‐1S allele from wheat has previously been reported as an IgE‐binding allergen [5, 6]. Some members of this protein family exhibit N‐linked glycosylation, and structural stability and resistance to enzymatic degradation have been described for 7S globulins, characteristics that may contribute to the preservation of their IgE‐binding capacity during food processing [5, 7–9]. These observations provided a rationale for further exploratory investigation of GLB1 but do not indicate that GLB1 is more clinically relevant than the other proteins identified in this patient.

Because the present patient had a history of severe buckwheat allergy, we further explored the sequence relationship between maize GLB1 and buckwheat Fag e 5. Fag e 5 is a vicilin‐like 7S globulin belonging to the cupin superfamily, with a molecular weight of ~50–60 kDa and partial glycosylation [10, 11]. It shares structural homology with other plant‐derived 7S globulins, such as peanut Ara h 1 and soybean Gly m 5 [12].

BLAST sequence analysis identified a significant local alignment involving GLB1 residues 367–509, with 60 of 144 aligned positions being identical (42% identity), 90 of 144 positive substitutions (62%), 17 of 144 gaps (11%), a bit score of 117, and an *E*‐value of 7 × 10^−36^ (Figure 3). This aligned region is located within the second cupin domain of GLB1. Within this broader aligned region, the 10‐amino‐acid sequence “FVVPAGHPFV” was completely conserved. This motif is presented as an example of sequence conservation within the aligned region and should not be interpreted as a known or predicted IgE epitope or as evidence of a biologically relevant cross‐reactive site.

**Figure 3:**
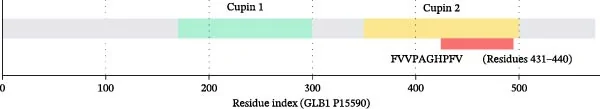
GLB1 (*Zea mays*) domain map showing the conserved sequence. Sequence alignment of maize GLB1 and buckwheat Fag e 5. Sequence comparison between maize GLB1 and buckwheat Fag e 5 was performed using BLAST. A significant local alignment involving GLB1 residues 367–509 showed 60/144 identities (42%), 90/144 positives (62%), and 17/144 gaps (11%), with a bit score of 117 and an *E*‐value of 7 × 10^−36^. The completely conserved 10‐amino‐acid sequence “FVVPAGHPFV” (GLB1 residues 431–440) within the second cupin domain is highlighted as an example of sequence conservation and is not intended to represent a demonstrated IgE epitope or cross‐reactive site.

The observed sequence similarity between maize GLB1 and buckwheat Fag e 5 is therefore hypothesis‐generating. Sequence similarity alone cannot establish immunological or clinical cross‐reactivity. Demonstration of cross‐reactivity would require additional functional experiments, such as IgE inhibition assays using buckwheat extract or purified or recombinant Fag e 5. Such experiments could not be performed in the present study. Therefore, our findings indicate sequence similarity between these proteins but do not demonstrate cross‐reactivity between maize GLB1 and buckwheat Fag e 5.

Another limitation concerns the relationship between the one‐ and two‐dimensional immunoblotting results. IgE‐reactive bands were observed at ~45 and 60 kDa in the one‐dimensional immunoblot. In the two‐dimensional analysis, GLB1 (theoretical molecular weight: 69 kDa) was identified from spot a. In contrast, the IgE‐reactive region b was observed at ~50–60 kDa and was separated into spots b‐1 and b‐2 based on the corresponding protein‐staining pattern. Mass spectrometry identified ketol‐acid reductoisomerase, chloroplastic (theoretical molecular weight, 55 kDa) in spot b‐1 and UDP‐glucose pyrophosphorylase 2 (theoretical molecular weight, 52 kDa), in spot b‐2. However, the ~45‐kDa IgE‐reactive band observed in the one‐dimensional immunoblot could not be unequivocally matched to a specific spot in the two‐dimensional immunoblot. The reason for this discrepancy remains unclear.

Clinically, this case highlights the importance of considering corn as a potential trigger in patients with suspected FDEIA. Immunoblotting combined with proteomic analysis may help identify candidate IgE‐binding proteins when conventional allergen testing does not fully characterize the responsible components.

This report has several limitations. First, this is a single‐case study without comparison groups, such as patients with immediate‐type corn allergy without exercise dependence or asymptomatic individuals sensitized to corn. Therefore, we cannot determine whether IgE reactivity to GLB1 or the other identified proteins is specific to corn‐dependent FDEIA or reflects corn sensitization more generally. Second, the identification of proteins by mass spectrometry from IgE‐reactive regions does not by itself establish their allergenicity or causal role in FDEIA. Third, functional studies directly demonstrating cross‐reactivity between maize GLB1 and buckwheat Fag e 5 were not performed. Further studies involving additional patients, appropriate comparison groups, and functional assays are required to clarify the allergenic relevance of the identified proteins and the potential immunological relationship between maize GLB1 and buckwheat Fag e 5.

In the present patient, GLB1 was identified as one of the three candidate IgE‐binding proteins and was selected for further hypothesis‐driven sequence analysis. These findings should be considered exploratory and require confirmation in additional patients.

## Author Contributions

Ayako Goto conceived the study, performed the literature review, and prepared the figures. Nobue Takamatsu conducted the experiments, performed the literature review, and prepared the figures. Tatsuya Moriyama and Yasuto Kondo provided critical advice on the study design and interpretation. Shinichiro Nagamitsu supervised the study and critically revised the manuscript.

## Funding

This study was supported by the JSPS KAKENHI (Grant JP23K01977).

## Disclosure

All authors read and approved the final manuscript.

## Ethics Statement

This study was approved by the Bioethics Review Committee of the Faculty of Medicine, Fukuoka University (Approval Number H21‐05‐005).

## Consent

Written informed consent for the publication of this case report and accompanying images was obtained from the patient’s parents.

## Conflicts of Interest

The authors declare no conflicts of interest.

## Data Availability

The data that support the findings of this study are available upon request from the corresponding author. The data are not publicly available due to privacy or ethical restrictions.
